# Oxidized low density lipoprotein and total antioxidant capacity in type-2 diabetic and impaired glucose tolerance Saudi men

**DOI:** 10.1186/1758-5996-6-94

**Published:** 2014-08-30

**Authors:** Essam Eldin Mohamed Nour Eldin, Abdullah Almarzouki, Adel Mohamed Assiri, Osman Mohammed Elsheikh, Badreldin Elsonni Abdalla Mohamed, Abdullatif Taha Babakr

**Affiliations:** Department of Medical Biochemistry, Faculty of Medicine, Umm Al-Qura University, Abdia, Makkah Saudia Arabia; Department of Internal Medicine, Faculty of Medicine, Umm Al-Qura University, Makkah, Saudia Arabia; Department of Biochemistry, Faculty of Medicine, International University of Africa, Khartoum, Sudan; Department of Biochemistry, Sciences Faculty for Girls, King Abdulaziz University, Jeddah, Saudia Arabia; Department of Medical Biochemistry, Faculty of Medicine, Umm Al-Qura University, Makkah, Saudia Arabia

**Keywords:** Oxidized LDL (Ox-LDL), Total antioxidant capacity, Prediabetics, Malondialdehyde (MDA)

## Abstract

**Background:**

Oxidative modification of low density lipoproteins (LDL) convert these native particles into pathogenic, immunogenic and atherogenic particles. Factors enhance LDL oxidation are poorly understood, especially in conditions of hyperglycemia. The present study was conducted to investigate which metabolic conditions are associated with the promotion of LDL oxidation in different glycemic situations.

**Methods:**

Adult male participants (274) were selected from patients admitted to the outpatient department of Diabetes Center in Al-Noor Specialized Hospital in Makkah and other citizens and residents in the city. The studied group was classified into three sub-groups: Group-I: control group of non-diabetic normal subjects, Group-II: subjects with impaired glucose tolerance (IGT) and Group-III: cases of type-2 diabetes mellitus (DM). Measurement of fasting blood glucose, 2 hour post-prandial blood glucose, glycosylated hemoglobin (HbA1c), triglycerides, serum cholesterol, HDL-cholesterol, LDL-cholesterol, ox-LDL, Total Antoxidant capacity (TAC) and Malondialdehyde (MDA) were performed. The obtained results were statistically analyzed.

**Results:**

Oxidation of native LDL increase nearly two folds in Type-2 DM group compared to controls. There is also significant increase in Ox-LDL of IGT group compared to controls. The correlation between Ox-LDL concentration and HbA1c in the whole population of the study confirms the increased Ox-LDL in subjects with hyperglycemia. A negative correlation exists between the concentration of Ox-LDL and total antioxidant capacity (TAC) in each studied group and in the whole population of the study as well. A positive correlation also exists between Ox-LDL concentrations and LDL values, more clear in controls and Type-2 DM, while this correlation was not significant in IGT group. The ratio of LDL oxidation as expressed by ox-LDL/LDL was increased in IGT group compared to control. More significant increase was observed in type-2 DM group.

**Conclusion:**

We concluded that the concentration of Ox-LDL increased in subjects with type-2 DM and IGT compared to controls. Moreover, oxidation of native LDL was associated with low levels of TAC and positively correlated with LDL levels, total cholesterol, HbA1c, body mass index (BMI) and increased age.

## Introduction

It is now widely accepted that oxidative modification of low density lipoproteins (LDL) convert these native particles into pathogenic [1], immunogenic [2, 3] and atherogenic [4, 5] particles. Current clinical research addresses the oxidation of LDL as a causative and initiating event in many pathological conditions and the oxidative modification of LDL enhances its atherogenicity [6].

Ox-LDLs are pathogenic particles, they have a number of biologic activitiesthat contribute to the process of atherosclerotic lesion formation and other diseases. Removal of Ox-LDL from circulating blood is a promising therapeutic strategy against atherosclerosis and many other diseases [7]. This goal cannot be achieved without a better understanding of the processes by which native LDL get oxidized.

In spite of the considerable knowledge and literature that support the correlation between circulating Ox-LDL and many pathologic conditions, to the best of our knowledge, there remains a gap regarding the biochemical status of the blood and the biological conditions of the body that enhance oxidation of native LDL. More understanding of the role of oxidation of lipoproteins may allow more rationally targeted diagnostic and therapeutic procedures in clinical applications.

The chemical composition of LDL makes these particles susceptible to oxidation by different lipid oxidants. The polyunsaturated acyl chains of cholesterol esters, phospholipids and triglycerides are vulnerable to oxidation, as is the sterol of free cholesterol and cholesterol esters.

The apolipoprotein B-100, made of 4536 amino acid residues, with many exposed tyrosines and lysines, which can be directly oxidized or modified by lipid oxidation products. The *in vivo* mechanism of LDLoxidation remains unclear and there are different mechanisms that may be responsible for the process. They are divided into enzymatic and non-enzymatic processes. The non-enzymatic process of modification involves free transition metal ions such as iron and copper, which are involved in catalyzing lipid peroxidation. The enzymatic process involves a number of different enzyme systems, such as lipoxygenases, myeloperoxidase which catalyses the formation of hypochlorousacid leading to the formation of acetylated LDL, NADPH oxidases, and nitric oxide synthases [8].

Although native LDL are exposed to all enzymatic and non-enzymatic oxidants, they are protected by a potent array of antioxidants in plasma. Moreover, some of these antioxidants are a part of the LDL composition. The LDL and other biomolecules are protected from free radical attack by the action of antioxidant capacity in the blood.

A healthy aerobic life is characterized by a steady formation of reactive oxygen species (ROS) and reactive nitrogen species (RNS), balanced by a similar rate of their consumption by an enzymatic and non-enzymatic, finely monitored, antioxidant system.

Since enzymatic and non-enzymatic antioxidants work in a network manner to exert their protective effects, no single antioxidant could represent the overall antioxidant status in plasma. Therefore, plasma antioxidant status isthe result of interaction and cooperation of various antioxidants. The concept of total antioxidant capacity (TAC) was developed considering the synergistic role of those antioxidants rather than the simple sum of individual antioxidant action [9].

Diabetes mellitus (DM) and impaired glucose tolerance (IGT) are associated with many complications including hypertension, renal failure and coronary artery diseases. Previous studies have revealed that the excess of cardiovascular events are observed in patients with type- 2 diabetes and that adequate control of Low Density Lipoprotein cholesterol has been proved to minimize the risk, however, lowering LDL will not be a complete solution. This is clear because at any given level of LDL concentration, there is great variability in the clinical expression of the disease. The atherogenic potential of diabetes could be associated with the modified proteins as the result of glycation and oxidation. The role of Ox-LDL as a residual lipid risk attracts considerable attention [10]. The situations of insufficient and/or inefficient insulin action were reported tocoincide with increased concentrations of the Ox-LDL [11], however, in which biochemical situation would these native LDL undergo oxidation is not well clarified.

The aim of the current study is to investigate the conditions where LDL undergoes oxidation and the possible role of the parameters of lipid profile (Total Cholesterol, Triglycerides, HDL and LDL) as well as the TAC, in increasing circulating Ox-LDL.

### Subjects and methods

This study was conducted in Makkah Al-Mukarama (KSA). The study protocol was approved by the Biomedical Ethics Committee, Faculty of Medicine, Umm Al-Qura University, Makkah, KSA. Ethical approval number 43-01071435.

Participants were selected according to inclusion criteria from patients admitted to the Outpatient department of Diabetes Center in Al-Noor Specialized Hospital (Makkah, KSA) and other residents in the city during the period from May 2012 up to July 2013. Subjects included are adult males, aged 18-55, live in Makkah and agreed to participate in the study. 274 volunteers were subjected to the investigations, they have been informed about the nature of the study and the expected risk and they have signed the ethical consent form. They have also filled out the structured questionnaire. Measurements of weight, height and blood pressure were performed by trained technicians, the Body Mass Index (BMI) was calculated as weight (Kg) divided by height squared (m^2^).

The studied group was classified into three sub-groups according to the American Diabetes Association (ADA) recommendations for diagnoses of diabetes and classification of glucose tolerance [12].

**Group-I:** Control group of non-diabetic normal subjects who met the following criteria:

Fasting blood glucose: <6.1 mmol/l (<110 mg/dl.) and

2-hour postprandial: <7.8 mmol/l. (<140 mg/dl.)

**Group-II:** Subjects with IGT who met the following criteria:

Fasting blood glucose: <7.0 mmol/l (<126 mg/dl.) and

2-hour postprandial: ≥7.8 mmol/l. (≥140 mg/dl.)

**Group-III:** cases of type-2 DM and subjects who met the following criteria:

Fasting blood glucose: ≥7.0 mmol/l (≥126 mg/dl.) and/or

2-hour postprandial: ≥ 11.2 mmol/l. (≥200 mg/dl.)

Not categorized as an Insulin Dependent Diabetes Mellitus (IDDM).

Participants who were excluded from the study were those of known history of coronary heart diseases (CHD) or cardiovascular complications of type-2 DM, known IDDM, those who are younger than 18 or older than 55 years old and those with familial hypercholesterolemia.

Information regarding exclusion criteria was obtained from the medical care provider and physicians, patient records or directly from the participants.

## Methods

### Blood collection and storage

Blood samples were drawn in ethylene diamine tetra acetic acid (EDTA)-containing vacationer tubes. For serum samples, blood was collected in plan tubes and left for 30 min, then centrifuged for 15 min at 3000 rpm and the serum samples obtained. The tubes were then properly labeled and sent directly to the biochemistry laboratory. Serum samples that were intended for long storage were kept in -80°C up to the date of analysis.

### Measurement of oxidized low density lipoproteins (Ox-LDL)

Ox-LDL was measured using the commercially available Mercodia Ox-LDL Competitive ELISA kit (Mercodia AB, Sylveniusgatan 8A, SE-754 50 Uppsala, Sweden), intended to be used for the *in vitro*quantitative determination of Ox-LDL in human blood plasma. MercodiaOx-LDL ELISA kits uses Holvoet et. al. monoclonal antibody, 4E6, which is specific for oxidatively modified LDL [13]. The 4E6 antibody is directed against a conformational epitope in the apoB-100 moiety of LDL that is generated as a consequence of aldehyde substitution of the lysine residues of apoB-100.

The principle of the procedure is based on the fact that Ox-LDL in the sample competes with a fixed amount of Ox-LDL bound to the microtiter well for the binding to the biotin-labeled specific antibodies 4E6. After a washing step that removes un-reactive sample components, the biotin-labeled antibody bound to the well is detected by HRP-conjugated streptavidin. After a second incubation and an additional washing step, the bound conjugate is detected by reaction with 3,3’,5,5’-tetramethylbenzidine (TMB). The reaction is stopped by adding acid to give a colorimetric endpoint that is read spectrophotometrically.

### Measurement of total antioxidant capacity (TAC)

For the assessment of TAC, we used the Cayman Antioxidant Assay Protocol. The assay can be used to measure the TACin the plasma, serum, urine, saliva, and/or cell lysates. Aqueous- and lipid-soluble antioxidants are not separated in this protocol which described first by Miller et al. [14], thus, the combined antioxidant activities of all vitamins, proteins, lipids, glutathione, uric acid, and others are assessed.

The principle of the assay relies on the ability of antioxidants in the sample to inhibit the oxidation of ABTS* (2,2-Azino-di-[3-ethylbenzthiazoline sulphonate]) to ABTS*^+^ by metmyoglobin. The amount of ABTS*^+^ produced can be monitored by reading the absorbance at 750 nm or 405 nm. Under the reaction conditions used, the antioxidants in the sample cause suppression of the absorbance at 750 nm or 405 nm to a degree which is proportional to their concentration. The capacity of the antioxidant in the sample to prevent ABTS* oxidation is compared with that of Trolox, a water-soluble tocopherol analogue, and is quantified as millimolarTrolox equivalents.

### Measurement of Malondialdehyde (MDA)

MDA was measured using TBARS assay kit (Cayman Chemical Company, Ann Arbor, MI, USA) for assaying lipid peroxidation in plasma, serum and urine.

The principle of the assay, based on the procedure of Dawn-linsley et al. [15], is that the MDA-TBA adduct formed by the reaction of MDA and thiobarbituric acid (TBA) under high temperature (90-100°C) is measured calorimetrically at 530–540 nm. Concentration of MDA was expressed in (μM MDA).

### Measurement of routine chemistry

Measurements of glucose, HbA1c, total cholesterol (TC), triglycerides (TG), high density lipoprotein (HDL) and LDL were done using the standard procedures and available commercial kits in a fully automated system (COBAS integra 400 plus).

Cholesterol CHOD-PAP, triglycerides GPO-PAP, LDL-C plus second generation, HDL-C plus third generation, reagents (Roche Diagnostics, Indianapolis, IN) were used on the Chemistry Analyzer to determine levels of TC, TG, LDL-cholesterol and HDL-cholesterol, respectively. All assays were done following the recommended procedures for instrument operation, calibration, quality control, and assay guidelines. The instrument was calibrated using calibrator for automated systems (Roche Diagnostics) for glucose, TCand TAG, and calibrator for automated systems lipids (Roche Diagnostics) for LDL-cholesterol and HDL-cholesterol.

Results were expressed for all parameters in mg/dl. Except for HbA1c, where it was expressed as percentage of glycosylated hemoglobin [16].

### Statistical analysis

Descriptive statistics and one way ANOVA were used to compare the concentration of Ox-LDL as well as the other metabolic parameters between the three groups. Pearson correlation and regression procedures were used to find correlation between Ox-LDL concentration and the different studied parameters. P value of < 0.05 was considered as statistically significant.

Degree of oxidation of LDL alone was analyzed in correlation with the different metabolic parameters and TAC in the three groups. All statistical methods were performed using SPSS for windows (version 20, SPSS Inc.).

## Results

After exclusion of cases according to our exclusion criteria, 274 successful cases were included in the study, categorized into three groups according to their fasting and 2 hours Post Prandial glucose result. Normal controls were 125 subjects, IGT group were 77 subjects and 72 subjects of Type-2 DM.

The IGT group showed an increase in the BMI compared to the other two groups.

Markers of hypertension, as expressed in Systolic Blood Pressure (SBP) and Diastolic Blood Pressure (DBP), were elevated in Type-2 DM group. The SBP and DBP in the control group tend to be in normal range values. Characteristics and glucose tolerance of the studied groups are summarized in Table 1.

**Table 1:** **Characteristics and glucose tolerance of the studied groups**

	Control	IGT	Type2 DM	***p*** ^a^	***p*** ^b^
	***n = 125***	***n = 77***	***n = 72***		
**Age (Years)**	34 ± 9	39 ± 10	42 ± 9	< 0.001	< 0.001
**BMI (kg/m** ^**2**^ **)**	28.3 ± 4.9	31.1 ± 5.1	30.2 ± 5.1	< 0.001	< 0.01
**SBP (mmHg)**	124 ± 15	129 ± 12	136 ± 13	< 0.05	< 0.001
**DBP (mmHg)**	82 ± 11	83 ± 8	89 ± 11	NS	< 0.001
**FBS (mg/dl.)**	91 ± 10	116 ± 21	193 ± 65	< 0.001	< 0.001
**2hrPP BS (mg/dl.)**	109 ± 18	167 ± 19	298 ± 79	< 0.001	< 0.001
**HbA1c%**	5.0 ± 0.6	6.7 ± 1.2	8.5 ± 2.1	< 0.001	< 0.001
**HbA1c mmol/mol**	31.1 ± 7.1	49.7 ± 12.8	69.4 ± 22.5	< 0.001	< 0.001

Results are expressed as mean ± SD.

***p*** ^**a**^: *p value* when control group was compared to IGT group.

***P*** ^**b**^: *p value* when control group was compared to Type2 DM group.

***NS:*** not significant.

*Abbreviations*: *BMI* Body mass index, *SBP* Systolic blood pressure, *DBP* Diastolic blood pressure, *FBS* Fasting blood sugar, *2hrPP* Two hour post-prandial blood sugar.

Normal lipid profile as expressed in terms of serum TC, TG, HDL-cholesterol, LDL-cholesterol and LDL/HDL ratio were observed in control group as shown in Table 2.

**Table 2:** **Lipids profile of the studied groups**

	Control	IGT	Type2 DM	***p*** ^a^	***p*** ^b^
	***n = 125***	***n = 77***	***n = 72***		
**CHOL. (mg/dl.)**	200 ± 53	240 ± 59	262 ± 79	< 0.001	< 0.001
**TG (mg/dl.)**	160 ± 78	213 ± 118	248 ± 158	<0.01	< 0.001
**HDL-C (mg/dl.)**	48.7 ± 16	53.2 ± 17	46.5 ± 16	NS	NS
**LDL-C (mg/dl.)**	118 ± 31	141 ± 38	153 ± 49	< 0.001	< 0.001
**LDL-C/HDL-C**	2.60 ± 0.9	2.92 ± 1.3	3.53 ± 1.4	NS	< 0.001

Results are expressed as mean ± SD.

***p*** ^**a**^: *p value* when control group was compared to IGT group.

***P*** ^**b**^: *p value* when control group was compared to Type2 DM group.

***NS:*** not significant.

*Abbreviations*: *CHOL* Cholesterol, *TG* Triglycerides, *HDL-C* High density lipoprotein cholesterol, *LDL-C* Low density lipoprotein cholesterol.

Oxidative stress parameters as shown in Table 3, demonstrated an increase in lipids peroxidation with decreased of TAC in IGT and type-2 DM groups compared to control group.

**Table 3:** **Oxidative stress parameters of the studied groups**

	Control	IGT	Type2 DM	***p*** ^a^	***p*** ^b^
	***n = 125***	***n = 77***	***n = 72***		
**Ox-LDL**	73.5 ± 27.5	108.7 ± 23.7	143.5 ± 21.9	< 0.001	< 0.001
**(U/L)**					
**MDA**	4.46 ± 1.98	5.77 ± 2.50	7.91 ± 3.17	< 0.001	< 0.001
**(μM)**					
**TAC**	1.384 ± 0.688	1.000 ± 0.397	0.817 ± 0.248	< 0.001	< 0.001
**(mM)**					
**Ox-LDL /LDL-C**	25.12 ± 11	31.81 ± 11	42.40 ± 25	< 0.01	< 0.001

Results are expressed as mean ± SD.

***p*** ^**a**^: *p value* when control group was compared to IGT group.

***P*** ^**b**^: *p value* when control group was compared to Type2 DM group.

*Abbreviations*: *Ox-LDL* Oxidized low density lipoprotein, *MDA* Malondialdehyde, *TAC* Total antioxidant capacity.

Oxidation of native LDL increased nearly two folds in the type-2 DM group compared to controls. The difference was significant, p < 0.001.

TAC was found to be low in IGT group compared to control. More decrease in TAC was found in Type-2 DM group p < 0.001. The ratio of LDL oxidation as expressed by Ox-LDL/LDL was increased in IGT and Type-2 DM groups compared to control as shown in Table 3.

### Correlations

Table 4 shows the correlations between Ox-LDL concentration and selected parameters in all the studied subjects.

**Table 4:** **Correlations between ox-LDL and selected parameters in the whole population of the study**

Parameter	Pearson correlation with Ox-LDL
**Age**	0.42**
**BMI**	0.21**
**FBS**	0.56**
**2hrPP BS**	0.62**
**Hb A1c**	0.60**
**CHOLESTEROL**	0.32**
**TRIGLYCERIDES**	0.16*
**LDL-C**	0.36**
**MDA**	0.44**
**TAC**	-0.51**
**OX-LDL-Abs**	0.59**
**LDL-C/HDL-C**	0.28**

*: significant at 0.01 level.

**: significant at 0.001 level.

Ox-LDL was found to be increased with age, the Pearson correlation coefficient (*r* = 0.42) in all subjects the correlation was significant (*p* < 0.001). BMI also showed a weak, but significant, positive correlation (*r* = 0.21) with Ox-LDL concentration in the studied group, *p* < 0.01.

**Figure 1:**
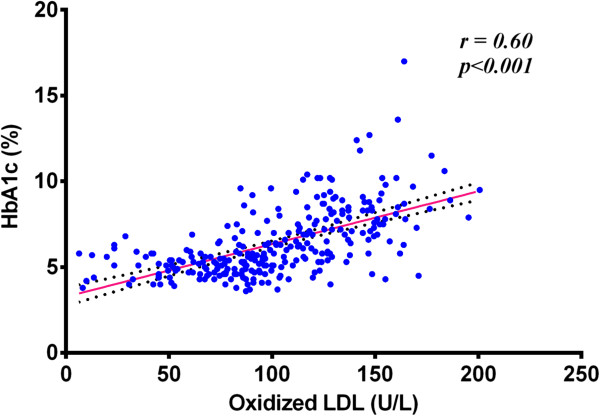
**Correlation between Ox-LDL and HbA1c in all studied groups.**

The correlation between Ox-LDL concentration and HbA1c in the whole population of the study confirms the increased Ox-LDL concentration in subjects with hyperglycemia. The Pearson correlation coefficient (*r* =0.60), as shown in Table 4, was found to be significant (*p* < 0.001). This correlation was shown in Figure 1.

**Figure 2:**
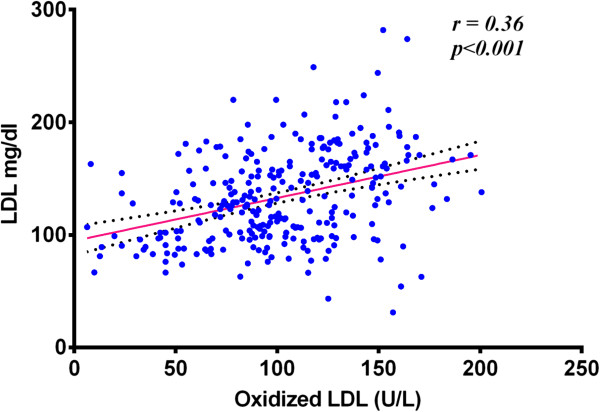
**Correlation between Ox-LDL and LDL in all studied groups.**

A positive correlation also exists, as shown in Table 4, between Ox-LDL concentrations and LDL values, more clear in controls, (*r* = 0.24) and type-2 DM (*r* =0.12), while this correlation is not significant in IGT group. In all subjects, Pearson correlation coefficient (*r* = 0.36) was found to be significant with (*p* < 0.001). as shown in Figure 2.

MDA levels were positively correlated with Ox-LDL levels in the study, Pearson correlation coefficient (*r* =0.44) was found to be significant as shown in Table 4.

**Figure 3:**
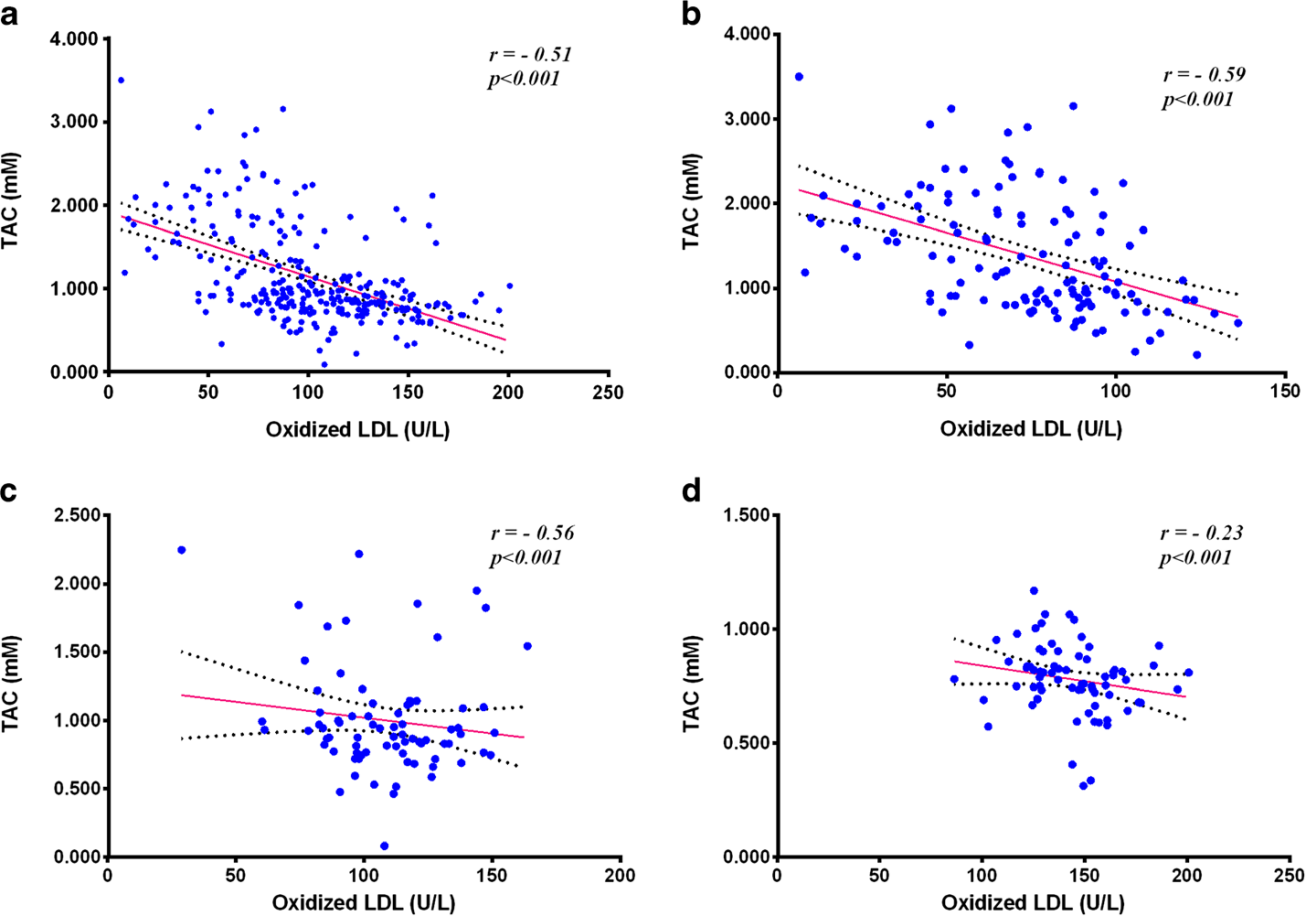
**Correlation between Ox-LDL and total antioxidant capacity. a**: in all studied groups. **b**: in control group. **c**: in IGT group. **d**: in type-2 DM group.

A negative correlation exists between the concentration of Ox-LDL and the concentration of TACin each group as well as in the whole population of the study.The correlation between the concentrations of Ox-LDL and TAC in the studied groupswas shown in Figure 3.

## Discussion

### Ox-LDL in diseases

Ox-LDL is attracting considerable importance in recent biomedical research. Oxidation of LDL and their subsequent uptake by macrophages inside the arterial wall are considered crucial steps in the process of atherosclerosis. Indeed, macrophages do not take up native LDL particles, which can be recognized by LDL receptor, unless they undergo *in vivo* modifications such as oxidation, where they are no longer been recognized in their normal metabolic pathway [1]. The process is considered as an initiating event of cardiovascular disease (CVD). According to many clinical studies, there is enough evidence that lowering LDL levels is an important prevention strategy for diabeticcardiovascular complications [17]. However, a residual risk of CVD tends to persist, this is suggested by many studies, at least partly, due to increased Ox-LDL levels [17, 18]. Moreover, targeting Ox-LDL was found to improve insulin sensitivity and immune cell function thereby reducing vascular inflammation in diabetic conditions in animal models [19]. Understanding the mechanisms and conditions that enhance LDL oxidation is of vital importance.

### Oxidative stress parameters and Ox-LDL

Oxidative stress is the imbalance between oxidants and antioxidants in the body caused by free radicals. Free radicals can be defined as reactive chemical species, mainly ROS and RNS, having a single unpaired electron in an outer orbit [20]. Oxidative stress is balanced by the body’s endogenous antioxidant systems and the ingestion of exogenous antioxidants.

The antioxidant defense system in the human body is a powerful system working in synergistic manner to provide what is known as TAC.

In the present study, there was an increase in lipid peroxidation with decreased TAC in IGT and type-2 DM groups compared to control group. Oxidation of native LDL in the present study increased by nearly two folds in type-2 DM group compared to control subjects. There is also significant increase in the mean Ox-LDL of group-II (IGT), compared to controls (*P* < 0.001). This increase can be read as a logical result of decreased antioxidant capacity observed in type-2DM and IGT groups.

TAC was significantly decreased (*P* < 0.001) in IGT group compared to control, more significant decrease (*P* < 0.001) was observed in type-2 DM group.

A negative correlation exists between the concentration of Ox-LDL and mM concentration of TAC in each studied group and in the whole population of the study as well. Pearson correlation coefficient was found to be (*r* = -0.59) as shown in Figure 3b, (*r* = -0.56) Figure 3c and (*r* = -0.23) Figure 3d, in controls, IGT and type-2 DM groups, respectively, while (*r* = -0.51) as shown in Figure 3a, in all the studied population (*P* < 0.001). Decreased TAC seems to play a vital role in the oxidation of LDL, this may explain the persistence of CVD risk even in case of controlled lipid and normal LDL concentration. We think that native LDL may undergo oxidation even when there is normal lipid profile and normal LDL concentration if there is low antioxidant defense in the body. The effect of high levels of LDL on the process cannot be neglected, however, the antioxidant status of the blood has the stronger effect according to our data.

Kopprasch et al. [21] suggest that Ox-LDL levels were not associated with the parameters of the oxidative/antioxidative balance in the blood. They reported that LDL cholesterol and triglycerides were the strongest predictors of circulating Ox-LDL levels, followed by HDL- cholesterol. They added that the strong correlation of Ox-LDL with LDL-cholesterol and TAG indicates that LDL oxidation in IGT is preferentially associated with dyslipidemia and that Ox-LDL increase may explain the high atherogenic potency of dyslipidemia in the pre-diabetic state [21].

Our study agrees with this positive correlation between Ox-LDL and dyslipidemia, however, it is not strong enough to exclude the effect of oxidative stress as suggested by Kopprasch et al. [21]. Furthermore, TAC has the major role in the oxidation of LDL with higher correlation coefficient than lipid profile parameters as suggested by our results.

The effect of antioxidant status on LDL oxidation was further studied for the possible effects of dietary supplementations with sources of antioxidant vitamins and polyphenols on antioxidant capacity and oxidation of LDL [22]. A decrease in Ox-LDL was reported after consumption of grape juice, a source of polyphenols [23], green tea [24], cocoa drink and cranberries [25, 26]. Vitamin E and C were studied as well as carotenoids in long-term supplementation and they were found to reduce *ex vivo* LDL oxidizability and *in vivo* lipid peroxidation [27, 28].

Lipid peroxidation as expressed in terms of MDA concentration was found to be significantly elevated (*P* < 0.001) in type-2 DM and IGT groups compared to control group.

Matsuda et al. [17] found that MDA-LDL level was significantly correlated with the levels of LDL-cholesterol, TG, HDL-cholesterol, but not with age, BMI, waist circumference, blood pressure, creatinine or HbA1c levels. Thus, they suggest that in addition to statin therapy, the management of dyslipidemic MS components is important for reducing the oxidization of LDL and, ultimately, the risk of cardiovascular events in high-risk DM patients [17].

### Ox-LDL and glycemic status

Diabetes is a group of metabolic diseases characterized by hyperglycemia resulting from defects in insulin secretion, insulin action, or both [29]. In Saudi Arabia, the overall prevalence of DM in adults is 23.7% [30]. The prevalence of diabetes is increasing, as a consequence of increasing incidence due to demographic changes such as aging, and as a result of risk factors such as obesity and sedentary life becoming more common [31]. In 2011 a study was conducted in KSA and revealed that the prevalence of DM type-2 in Saudi Arabia has increased by a whopping 10.0% in just a decade [32]. This may give a strong justification for studies, such as the present study, dealing with the etiology and mechanism of complications associated with diabetes and other situations of hyperglycemia.

The present study investigates parameters of dyslipidemia and oxidative stress in correlation to Ox-LDL in three groups of different glycemic situations.

In the present study, Ox-LDL levelsare higher in IGT and type-2DM subjects when compared to control *p* < 0.001. Higher levels of Ox-LDL were observed in diabetics suggesting that oxidation of LDL is positively influenced by hyperglycemia. In contrast, Schwenke, et al. [33] found that glycemic status negatively influenced LDL oxidizability, with a paradoxical reduction in LDL oxidizability, as indicated by a lower LDL oxidation rate with increased extent and duration of glucose intolerance. They added that the difference was only slightly attenuated by adjustment for relevant demographic, metabolic, dietary, and pharmacological factors that potentially influence LDL oxidation [33].

Previous studies suggest that serum levels of Ox-LDL were significantly increased in IGT versus NGT subjects [21]. Our results show increased oxidation of native LDL in cases of IGT.Ox-LDL is positively correlated with FBS levels in the studied group, (r = 0.56) and (*P* < 0.001). A stronger positive correlation observed between Ox-LDL and the 2hPP values was also significant, (r = 0.62) and (*P* < 0.001). These findings suggest that oxidizability of native LDL is higher in hyperglycemia. The positive correlation between Ox-LDL concentration and HbA1c in the whole population of our study confirms the increased Ox-LDL concentration in subjects with hyperglycemia. The Pearson correlation coefficient (*r* = 0.60) was found to be statistically significant *P* < 0.001.

### Effect of age and BMI on the oxidation of LDL

Those who were 55 or older were excluded from the study because of the known increased oxidative stress in elderly people [34, 35], which may give misleading results.

The diabetic and IGT groups were older in age in comparison with the normal healthy group, as shown in Table 1, aconstant observation in previous studies as stated by Li, Saito et al. [36], old age is a non-modifiable risk factor for CVD and other diseases related to aging, IGT and type-2 diabetes are correlated with older ages. In the present study,Ox-LDL was found to be increased with age, the Pearson correlation coefficient (*r* = 0.422) between age and Ox-LDL in the whole subjects was found to be significant*P* < 0.001. This may be as a result of decreased antioxidant capacity observed in older people in previous studies and in the present study as well.

The BMI was significantly increased in type-2 DM group (*P* < 0.05) and more significantly increased in the IGT group (*P* < 0.001).

So many studies consider obesity as a strong risk factor of type-2 diabetes, its cardiovascular complications and independent risk factor of CVD [37]. Our results in the present study show a significant positive correlation between BMI and Ox-LDL concentration (*p* < 0.01). This may explain the increased prevalence of type-2 DM and cardiovascular complication observed in obese subjects by many previous studies [38]. The strong link between Ox-LDL and risk of CVD was well established, and even their vital role in foam cell formation. Our results indicate higher concentrations of Ox-LDL in obese cases.

### Ox-LDL and lipid profile

Dyslipidemia is strongly linked to insulin resistance and other features of the metabolic syndrome. Diabetic dyslipidemia is a well-recognized risk factor for atherosclerotic CVD, and lipid lowering drugs proved an effective treatment for decades [39]. Ox-LDL concentration was found to be positively correlated with TC levels in the studied group (r = 0.32) and (*P* < 0.001). A weak positive correlation was also observed with TG levels. Diwadkar et al. [40] suggest that elevated levels of serum TG may contribute to the rapid oxidation of LDL seen in diabetic subjects [40].

**Figure 4:**
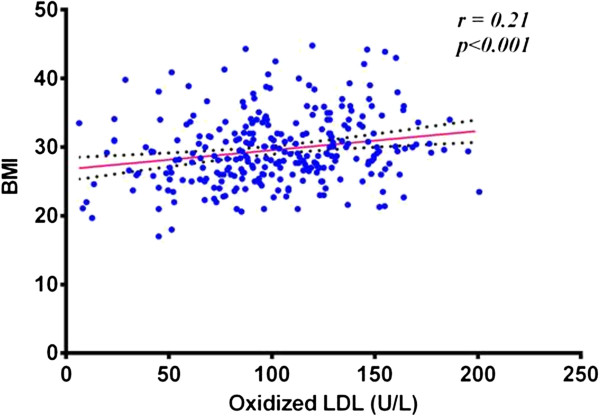
**Correlation between Ox-LDL and BMI in all studied groups.**

A positive correlation also exists between Ox-LDL concentrations and LDL values, more clear in controls (*r* = 0.24) and type-2 DM (*r* = 0.12), while this correlation is not significant in IGT group. In all subjects, Pearson correlation coefficient (*r* = 0.36) was found to be significant with *P* < 0.001. Our data suggest that oxidation of LDL increase in case of dyslipidemia and the oxidation of these particles particularly associated with high levels of native LDL. This may be handled as a logical conclusion in that, if there is more native LDL then there should be more Ox-LDL. However, we think that it is a whole situation of dyslipidemia that enhances LDL oxidation. Sedentary lifestyle and obesityprovide a good chance for disturbance in lipid profile and consequently oxidation of LDL and related cascade of events. Ox-LDL concentration in the present study was found to be positively correlated with BMI as shown in Figure 4.

Our findings are also supported by the results reported by Magge, et al. [41] who concluded that obese pre-diabetic adolescents have a significantly more atherogenic lipoprotein profile compared with obese normglycemic peers [41]. Another study reported that the diabetic patients with poor glycemic control exhibited a significant increase in cholesterol and TG and decrease in HDL levels [42]. Therefore, the present study suggest that management of obesity and dyslipidemia, which are more common among Saudi office workers than general population according to previous studies [43], may help in prevention of LDL oxidation and manage further complications caused by the increased Ox-LDL.

### The ratio of LDL oxidation

The ratio of LDL oxidation as expressed by Ox-LDL/LDL was increased in IGT group compared to the normal control (*P* < 0.01). More significant increase was observed in type-2 DM group (*P* < 0.001). Thus, our data suggest that not only does the concentration of Ox-LDL increase, but also LDL oxidizability increases in diabetic subjects and in cases of IGT.

## Conclusion

In conclusion, our study has found that Ox-LDL levels increase in IGT and type-2 DM subjects when compared to control.

Oxidation of LDL is negatively correlated with the TAC of blood. A positive correlation between Ox-LDL and concentration of native LDL was also found, however, low antioxidant capacity play the major role in LDL oxidation.

More studies are recommended to investigate whether increasing the antioxidant capacity either by dietary or medical intervention routes will prevent or reduce oxidation of LDL.

This study also shows a positive correlation between BMI and elevated levels of Ox-LDL. Weight control is recommended to avoid the increase in atherogenicOx-LDL particles.

### Limitations of the study

The study included only Saudi male participants, other studies including women and different ethnic groups are needed to confirm these findings.

The study measure antioxidants as a total sum. Measuring dietary and enzymatic antioxidants in similar studies may be beneficial.

## Authors’ informations

1. EMN: MBBch (Cairo University), Msc (Medical Biochemistry), PhD (Clinical Biochemistry), X-Dean of Faculty of Medicine, Zagazig University (Egypt), Professor of Clinical Biochemistry, Faculty of Medicine, Umm Al Qura University, KSA.
2. AA: Graduate at Vienna University, college of Medicine (Dr.Med.Univ.), Facharzt in Internal Medicine, (University Hospital Vienna). Fellowship Endocrinology & Metabolism, Austrian Medical Association. Fellow of Austrian medical Association (F.AM.A), Assistant professor of internal medicine, X-Dean of Faculty of Medicine, Umm Al Qura University, KSA.
3. AMA: B.Sc Faculty of Medicine and applied Science, University of King Abulaziz ,KSA. Ph.D. Clinical Biochemistry, Faculty of Medicine, Scheffield University. Professor of Biochemistry, Faculty of Medicine, Umm Al Qura University, KSA. Head of the the Institute of Scientific Research and Revival of Islamic Heratige, Umm Al-Qura University, Makkah, KSA.
4. OME: BSc. Biochemistry, Ain Shams University, Egypt. MSc. PhD. Biochemistry, University of Gezira, Sudan. Associate Professor of Biochemistry, International University of Africa, Sudan. Associate professor of Biochemistry, Shagra University, KSA.
5. BEA: BSc. Biochemistry, Khartoum University, Sudan. MSc. PhD. Biochemistry, University of Gezira, Sudan. Associate professor of Biochemistry and X-Head Department of Biochemistry and nutrition, Faculty of Medicine, University of Gezira, Sudan. Associate professor of Biochemistry, Faculty of Science, King Abdulaziz University, KSA.
6. ATB: BSc. Biochemistry, Alexandria University, Egypt. MSc. Biochemistry, Faculty of Basic Medical Sciences, Omdurman Islamic University, Sudan. Lecturer of Clinical Biochemistry, Faculty of Medicine, Umm Al Qura University, KSA.

## Abbreviations

2hrPP: Two hours Post Prandial
ABTS: Azino-di-[3-ethylbenzthiazoline sulphonate]
ADA: American Diabetes Association
APO: Apoprotein
BMI: Body mass index
CHD: Coronary heart diseases
CVD: Cardiovascular disease
DBP: Diastolic blood pressure
EDTA: Ethylene diamine tetra acetic acid
ELISA: Enzyme linked immunosorbent assay
FBS: Fasting blood sugar
HBA1c: Glycated hemoglobin
HDL: High density lipoproteins
IC: Immune complx
IDDM: Insulin dependent diabetes mellitus
MDA: Malondialdehyde
NGT: Normal glucose tolerance
Ox-LDL: Oxidized low density lipoprotein
RNS: Rreactive nitrogen species
ROS: Reactive oxygen species
TAC: Total antioxidant capacity
TBA: Thiobarbituric acid
TBARS: Thiobarbituric acid reactive species
TC: Total cholesterol
TG: Triglycerides
TMB: Tetramethylbenidine
TOH: Tocopherols
VLDL: Very low density lipoproteins.
